# Deletion of Fifteen Open Reading Frames from Modified Vaccinia Virus Ankara Fails to Improve Immunogenicity

**DOI:** 10.1371/journal.pone.0128626

**Published:** 2015-06-08

**Authors:** Naif Khalaf Alharbi, Alexandra J. Spencer, Adrian V. S. Hill, Sarah C. Gilbert

**Affiliations:** 1 The Jenner Institute, University of Oxford, Oxford, OX3 7DQ, United Kingdom; 2 King Abdullah International Medical Research Center, Riyadh, Saudi Arabia; University of Iowa, UNITED STATES

## Abstract

Modified vaccinia virus Ankara (MVA) is a highly attenuated strain of vaccinia virus, which has been used as a recombinant vaccine vector in many vaccine development programmes. The loss of many immunosuppressive and host-range genes resulted in a safe and immunogenic vaccine vector. However it still retains some immunomodulatory genes that may reduce MVA immunogenicity. Earlier reports demonstrated that the deletion of the *A41L*, *B15R*, *C6L*, or *C12L* open reading frames (ORFs) enhanced cellular immune responses in recombinant MVA (rMVA) by up to 2-fold. However, previously, we showed that deletion of the *C12L*, *A44L*, *A46R*, *B7R*, or *B15R* ORFs from rMVA, using MVA-BAC recombineering technology, did not enhance rMVA immunogenicity at either peak or memory cellular immune responses. Here, we extend our previous study to examine the effect of deleting clusters of genes on rMVA cellular immunogenicity. Two clusters of fifteen genes were deleted in one rMVA mutant that encodes either the 85A antigen of *Mycobacterium tuberculosis* or an immunodominant H2-K^d^-restricted murine malaria epitope (pb9). The deletion mutants were tested in prime only or prime and boost vaccination regimens. The responses showed no improved peak or memory CD8^+^ T cell frequencies. Our results suggest that the reported small increases in MVA deletion mutants could not be replicated with different antigens, or epitopes. Therefore, the gene deletion strategy may not be taken as a generic approach for improving the immunogenicity of MVA-based vaccines, and should be carefully assessed for every individual recombinant antigen.

## Introduction

Modified Vaccinia virus Ankara (MVA) is a highly attenuated strain of vaccinia virus, the smallpox vaccine. This attenuation was achieved by more than five hundred passages in chick embryo fibroblast (CEF) cells [1]. The resultant MVA is incapable of replication in almost all tested mammalian cells, except the baby hamster kidney cell line (BHK-21) [2]. MVA is safe virus that was used to vaccinate 120000 people during the WHO smallpox eradication initiative in 1970s without adverse events [3]. The first clinical use of recombinant MVA (rMVA) as a vaccine against a human pathogen was in 2000. The safety profiles of the recombinant vaccine were as expected with a replication-deficient virus, and no live or dead virus (screened by PCR) could be detected in samples from the site of inoculation [4]. In the last decade, MVA-based vaccines have been tested in an increasing range of animal models and many clinical trials for vaccines against malaria, HIV\AIDS, Influenza, TB [5–7], and now Ebola (manuscript in preparation). The attenuation of MVA resulted in the loss of almost a third of its parental genome, deleting or mutating the majority of immune evasion and host range genes [8]. By losing immunomodulatory genes, MVA has become a strong immune activator, infecting a wide range of immune cells and eliciting a greater range of chemokines and cytokines compared to the parental vaccinia virus [9, 10]. However, it has retained some genes that are involved in the host-virus interaction and immune evasion such as *B15R*, encoding the IL-1β binding protein [11], or *A41L*, encoding a CC-chemokine binding protein [12].

It has been reported that deletion of some of these genes improved innate, adaptive memory cellular or humoral immune responses to the vector (MVA) or to the encoded recombinant antigens, and enhanced the protective capacity of MVA following a challenge with VACV WR. The deletion of the ORF *A41L [12]*, *B15R[11]*, or both [13], showed around 2-fold increase in the memory CD8^+^ T cell responses upon stimulation with VACV-infected cells or MVA-specific peptides. The immune responses to the recombinant antigens were also increased to a similar extent. In the double deletion ΔA41L\B15R [13], the MVA vector encoded HIV-1 clade B immunogen (MVA-B), and was delivered by DNA-prime MVA-boost immunization. The use of different peptide pools to stimulate the splenocytes from vaccinated mice revealed an increased CD8^+^ T cell response to one of the pools (GPN-pool) in the rMVA mutant vaccinated group. This mutant also induced more CD8^+^ T cells than CD4^+^ T cells in the Env-pool. In another study, the deletion of *C6L* gene from MVA-B (ΔC6-MVA-B) increased memory CD4^+^ and CD8^+^ T cell responses, and although this mutant did not shift the immune responses towards particular peptide pools, the increased CD4^+^ or CD8^+^ T cells were only observed in one peptide pool (the GPN-pool), but not in the Env- or Gag-pool [14]. Although the ΔC6-MVA-B was not tested for the peak (acute phase) adaptive immunity, a recent study from the same research group testing the effect of a double-deletion mutant lacking *C6L* and *K7R* in the same MVA-B vaccine (ΔC6/K7-MVA-B) showed that the ΔC6-MVA-B, included as a control, had no impact on the peak immune responses [15]. This is perhaps not surprising as the majority of the previous studies reported small (around 2-fold) or no enhanced immunogenicity of MVA deletion mutants at the acute adaptive phase. The double-deletion ΔC6/K7-MVA-B showed increased memory CD8^+^ T cells, but not CD4^+^ T cells; and upon comparison with the ΔC6-MVA-B, the increase seemed to be mainly an effect of C6 absence and not due to the *K7R* deletion [15]. Furthermore, in more recent studies, increased memory CD8^+^ T cells specific to the MVA vector or to encoded antigens were also reported upon the single deletion of *F1L [16]*, *N2L [17]*, or *C12L* (MVA008L) [18] in rMVA with around 2-fold increase. Overall, it appears that the increased cellular immunogenicity is antigen-specific, or rather epitope specific, which could make it difficult to employ the same deletion in a different MVA-based vaccine before carefully assessing the immunological outcome, and the detectable increase in immunogenicity is always small.

In terms of improving humoral immunogenicity in these studies, while the deletion of *A41L*, *B15R*, or the double deletion of both had no impact on the antibody induction at the memory phase, the deletion of *C6L* or *N2L* increased the memory antibody-mediated responses by around 2-fold in the total anti-recombinant antigen antibody titres. Of note, the *C6L* deletion enhanced the anti-gp120 antibody titres [14], but the re-testing of the *C6L* deletion mutant, in a later study, along with the double deletion *C6L/K7R* mutant showed no significant difference in the anti-gp120 antibody titres by those two mutants compared to the control (MVA-B vaccine) [15]. The humoral responses were not shown in the F*1L* and *C12L* studies. Finally, the deletion of *A35R* showed small non-significant increase in the cellular immunogenicity but increased the anti-VACV antibody titres by 2-fold with isotype switching to more IgG and its subclasses IgG1 and IgG2a [19]. Overall, in murine studies, it seems that all reported increases in the immunogenicity of MVA mutants are small, with 2- to 3-fold at most. In these reports there are many important variables such as the dose and route of administration, vaccination regimens (prime only or prime-boost), recombinant antigens, immunological readouts (e.g. ELISpot vs. ICS), mouse strains, time and type of stimuli used in the *ex vivo* re-stimulation (e.g. VACV-infected cells vs. E3 or F2(G) peptides), and the time for harvesting the spleens to determine memory responses (varies from 10, 21, 56, or 180 days post-MVA injection).

Nevertheless, a study in macaques reported that deleting four genes; *C12L* (MVA008L), *B15R* (MVA184R), *A41L* (MVA153L), and *A46R* (MVA159R) from MVA encoding HIV-1 clade C consensus Gag and Env immunogen, given twice with a 9 week interval, resulted in increased CD4^+^ and CD8^+^ T cells either after the prime (4-fold) or after the boost dose (5-fold); and around 25-fold increase in the anti-gp120 antibody titres [20]. In this report, the deletion of uracil-DNA-glycosylate (*udg*) gene, in addition to those four genes, resulted in a five-gene deletion rMVA mutant that did not further enhance the improved immunogenicity of the four-gene deletion mutant. The deletion of the fifth gene, *udg*, was expected to improve the immune responses, as a previous report by the same group showed that the deletion of *udg* gene alone in rMVA inhibited the late MVA gene expression, reduced the antigen complexity of MVA, and improved the elicited CD4^+^ and CD8^+^ T cells (2-fold) in a similar regimen, but with Gag gene from HIV clade B, which is a different recombinant antigen [21].

Previously, we reported that the single deletion of *C12L*, *A44L*, *A46R*, *B7R*, or *B15R*, from MVA did not enhance the cellular immunogenicity of the TIP model antigen (described in material and methods) using seven immunodominant MVA epitopes in two strains of mice at the peak cellular responses. Of the memory responses (day 56 post-MVA injection), only the ΔB15R-MVA showed a small (1.5-fold) but significant increase in the CD8^+^ T cell frequencies [22]. Here, we have extended our previous study using MVA-BAC recombineering technology to examine the effect of deleting clusters of genes (described in Table 1), including a large deletion of 15 genes in one mutant, on the cellular immunogenicity of r MVA. These deleted genes have different immunomodulatory functions or, in some instances, unknown functions. The derived recombinant MVA mutants (rMVA mutants) encode either the 85A antigen of *M*. *tuberculosis* [23] or the TIP model epitope string-based antigen [24] that encompasses an immune dominant H2-K^d^-restricted murine malaria epitope (pb9) from the *Plasmodium berghei* circumsporozoite protein [25]. The MVA-BAC system was shown previously to have no altering effect on the immunogenicity of recombinant MVA, and elicit anti-vector immunogenicity similar to the conventionally derived recombinant MVA [22].

**Table 1:** MVA deletion mutants.

MVA	Deleted ORF / Function (Reference)	Note
**MVA*wt***	None	Not recombinant virus. Not modified virus. Used as a control.
**MVA85A**	None	Conventionally derived rMVA with 85A at the TK^a^. Used as a control for 85A responses.
**MVA-BAC-85A**	None	BAC recombineering-derived rMVA with 85A at the TK^a^ locus. Contains BAC DNA and expresses GFP. Used as a control for 85A responses.
**MVA-TIP**	None	Contains BAC DNA, expresses GFP and encodes TIP antigen at TK^a^ locus. Used as a control for TIP responses.
**ΔC11-K3/B15-MVA**	*C11R* / viral growth factor [32]. *C10L* / suggested IL-1 binding protein [33]. *MVA008R*, not in VACV COP^b^, but *C12L* in WR^c^ ^)^/ IL-18 binding protein [34]. *D7L* / RNA polymerase subunit [35]. *MVA007R*, and *009L*-0*13L* (fragmented, not in VACV COP^b^) / cowpox host range genes [8]. *C9L* / (fragmented gene) [8]. *C8L* / unknown. *C7L* / human cells host range gene [36, 37]. *C6L* / anti-IFN-β activation (IRF3/IRF7 inhibitor and Bcl-2 family) [38]. *N1L* / anti-apoptotic (Bcl-2 family) [39]. *N2L* / anti-IFN-β activation (IRF3 inhibitor and Bcl-2 family) [40]. *K1L* (inactive gene) / human cells host range gene [41]. *K2L* / cell fusion (serine protease) inhibitor [42]. *K3L* / anti-apoptotic (PKR inhibitor) [43]. *B15R* ^d^ / IL-1β binding protein [11].	Contains BAC DNA, expresses GFP, and encodes TIP antigen at TK.
**ΔB7-15-MVA**	*B7R* / TNF-α soluble receptor and chemokine binding protein [44]. *B8R* (fragmented) / IFN-γ soluble receptor [45]. *B9R* / intracellular protein [46]. *B10R* / unknown. *B11R* / unknown. *B12R* / serine/threonine protein kinase[47]. *B13R*+*14R* (fragmented). *B15R* ^d^ / IL-1β binding protein [11].	Contains BAC DNA, expresses GFP, and encodes TIP antigen at TK.
**ΔA41-44/B15-MVA**	*A41L* / CC-Chemokine binding protein [12]. *A42R* / Profilin homolog [48]. *A43R* / membrane protein [49]. SalF6R (*MVA156R*) / membrane glycoprotein [50, 51]. *A44L* / Hydroxysteroid deydrogenase [51]. *B15R* ^d^ / IL-1β binding protein [11].	Contains BAC DNA, expresses GFP, and encodes TIP antigen at TK.
**ΔA41-46/B15-MVA**	Same as above, in addition to *A45R* / Inactive superoxide dismutase [52] *and A46R* / TLR signalling inhibitor [53].	Contains BAC DNA, expresses GFP, and encodes TIP antigen at TK.
**Δ15-MVA-TIP andΔ15-MVA-85A (i.e.Δ*A41L* to *46R* & *B7R* to *B15R*)**	*A41L*, *A42R*, *A43R*, *A44L*, *A45R*, *A46R*, *B7R*, *B8R*, *B9R*, *B10R*, *B11R*, *B12R*, *B13R*, *B14R*, *and B15R* ^d^ ^.^ In addition to *SalF6R*, which is located between *A42R* and *A43R*. All mentioned above.	Contains BAC DNA, expresses GFP, and encodes TIP antigen at TK.

MVA deletion mutants used in this study. Different clusters of ORFs were deleted from the mutants, encoding proteins with different functions (mentioned with references). In a few cases, there were no known functions associated with ORFs. The mutants express the 85A or TIP antigens that were inserted at the TK locus. All mutants were made using MVA-BAC recombineering, therefore, all contain BAC DNA and GFP (green fluorescent protein) marker.

^a^TK: Thymidine kinase locus, used as an insertion site for the recombinant antigens.

^b^VACV COP: Vaccinia virus Copenhagen strain.

^c^WR: Vaccinia virus Western Reserve strain.

^d^The ORF *B15R* is the *MVA184R* gene. It is named *B15R* in VACV WR while it is *B16R* in VACV COP. This made inconsistency in reporting this ORF as well as the downstream B fragment ORFs in the literature. Here, we report *MVA184* as the *B15R*, consistent with our previous work [22], and in accordance with another report *[11]*. However, it was reported as *B16R* in another study [13]. This ORF encodes IL-1β binding protein [11].

None of the derived rMVA mutants showed improved immunogenicity either to the MVA or to encoded TIP antigens. The large MVA deletion mutant (lacking fifteen genes) was tested in prime only or in prime-boost regimens and showed no improved CD8^+^ T cell frequencies. This unchanged immunogenicity was observed using either the synthetic model TIP antigen or the naturally occurring 85A antigen. The responses at 28, 56, or 84 (long-term) days post-MVA injection showed no improved memory CD8^+^ T cell frequencies, except to one peptide (E3) at day 28. However, at day 84, or at day 56 in the prime-boost vaccination, this increase in E3-specific responses was very marginal and not statistically significant. This support the earlier studies showing that the increase could be observed with only few peptides or peptide pools.

Our results suggest that the reported increases in MVA deletion mutants, which were small increases in many cases (around 2-fold), could not be replicated with different antigens and that the approach of gene deletion to improve MVA-vectored vaccines should be carefully assessed for every recombinant antigen rather than taken as a generic approach for improving MVA immunogenicity.

## Results

### The immunogenicity of recombinant MVA mutants with TIP antigen

**Fig 1:**
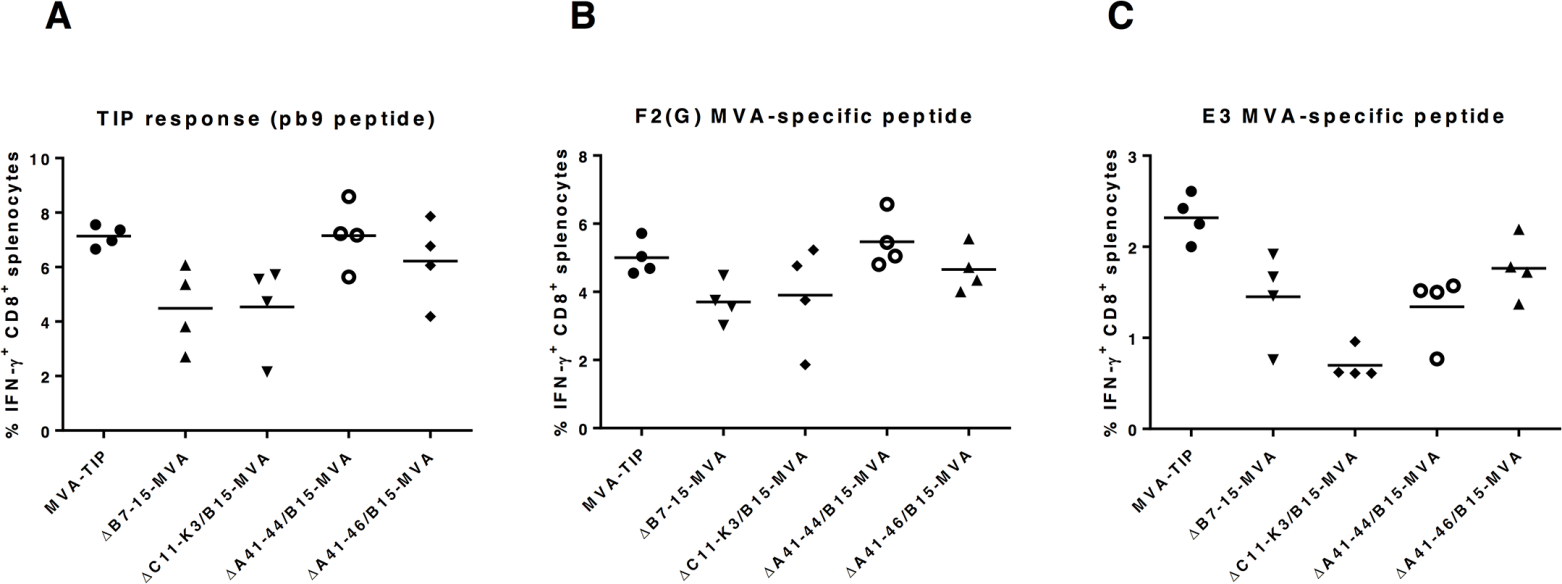
*In vivo* cellular immunogenicity of MVA deletion mutants, lacking clusters of genes, with TIP antigen. Five groups of female BALB/c mice (n = 4) were immunized (i.d.) with MVA*wt* with TIP model antigen (MVA-TIP), or MVA deletion mutant (lacking different clusters of genes, described in Table 1) at the dose of 1x10^6^ pfu/ml. **Seven days** post immunization, mice were sacrificed and spleens collected for intracellular cytokine staining and flow cytometry to determine the percentage of IFN-γ-secreting CD8^+^ T splenocytes in response to *in vitro* re-stimulation with pb9 peptide **(A)**, or with MVA vector-specific peptides **(B and C)**. These values are presented after subtracting the values of unstimulated cells for every mouse (sample). The mean of each group is shown. Data is representative of two independent experiments. There was no statistical significant increase in any of the tested groups as compared to the control (MVA-TIP) using Kruskal-Wallis test with Dunn's multiple comparisons.

To investigate the effect of deleting clusters of genes on the immunogenicity of a recombinant MVA, expressing TIP antigen, we immunized BALB/c mice with the recombinant MVA control (MVA-TIP) or with different recombinant MVA mutants (ΔB7-15-MVA, ΔA41-44/B15-MVA, ΔA41-46/B15-MVA, and ΔC11-K3/B15-MVA, from which a number of ORFs had been deleted, described in Table 1) intradermally (i.d.) and harvested spleens a week later. The IFN-γ-secreting CD8^+^ T cells, specific to MVA-peptides (E3 and F2(G)) or to the TIP-specific peptide (pb9) were then measured. There were no significant differences in response to the MVA-E3, MVA-F2(G), or pb9 peptide between any of the mutant groups and the control (Fig 1).

### The construction and sequence screening of a recombinant MVA mutant lacking fifteen genes

As there was no improved immunogenicity with various deletion mutants, we constructed a large MVA mutant, lacking fifteen genes (Δ15-MVA) described in Table 1, and investigated its impact on the cellular immunogenicity. We made two version of the Δ15-MVA with either 85A or TIP recombinant antigens (Δ15-MVA-TIP and Δ15-MVA-85A). The Δ15-MVA-85A was based on Δ15-MVA-TIP and was sent for genomic sequencing. The genome sequencing showed that MVA genome remained unchanged, despite passing through four rounds of MVA-BAC recombineering. Sequencing was performed on BAC DNA as well as on viral DNA, from a BAC-rescued virus, and revealed the presence of the two large deletions (*A41L* to *A46R* and *B7R* to *B15R*). The presence of either 85A or TIP antigens were screened with PCR; and the absence of the MVA wild type was also confirmed by PCR.

### The immunogenicity of a recombinant MVA mutant lacking fifteen genes, with TIP antigen

**Fig 2:**
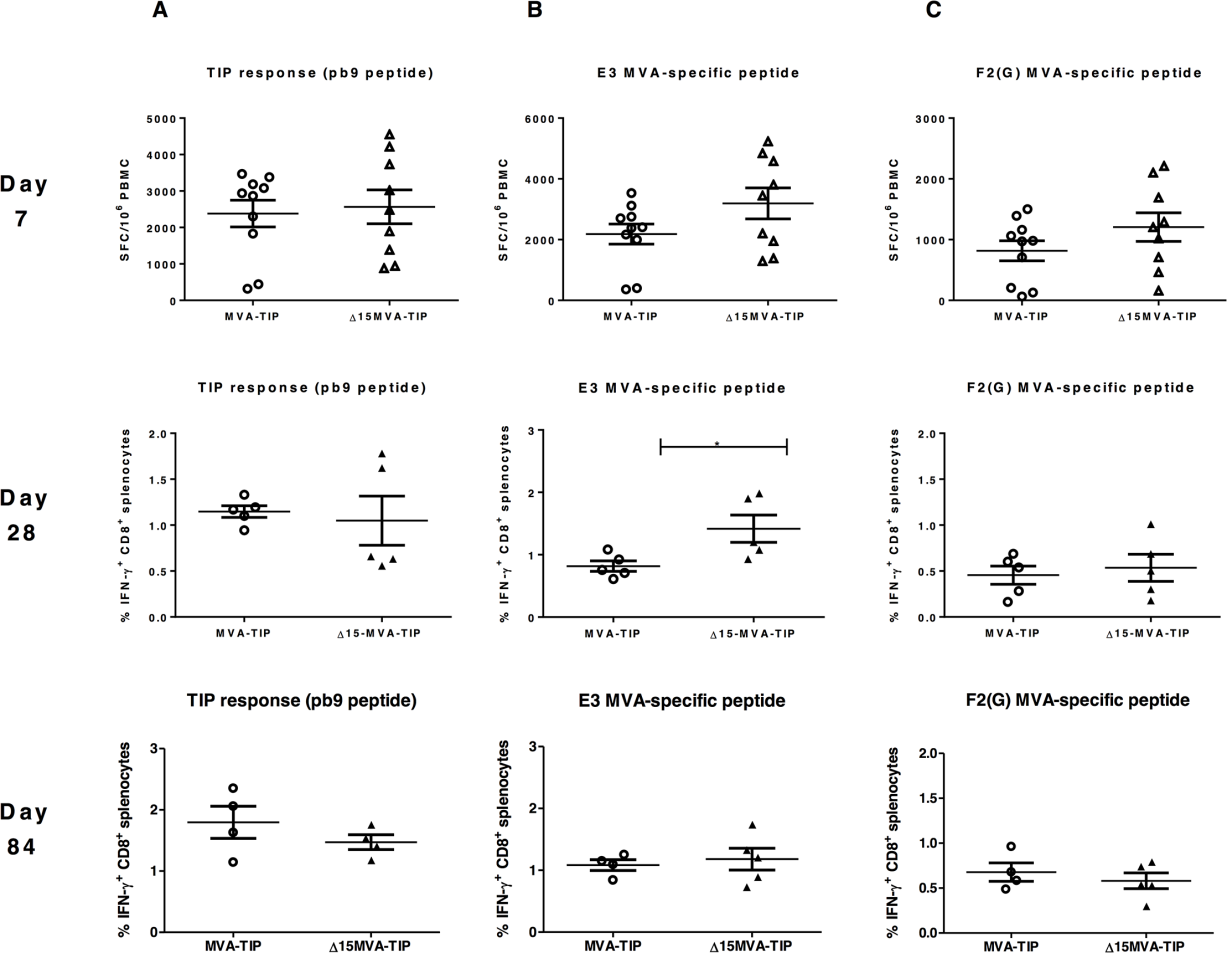
*In vivo* cellular immunogenicity of MVA deletion mutants, lacking fifteen genes, with TIP antigen. Two groups of female BALB/c mice (n = 10) were immunized (i.m.) with MVA*wt* with TIP model antigen (MVA-TIP), or MVA deletion mutant (lacking 15 genes) ∆15-MVA-TIP at the dose of 1x10^6^ pfu/ml. **Seven days** post immunization (top), *ex vivo* ELISpot was performed to determine the percentage of IFN-γ-secreting CD8^+^ splenocytes in response to *in vitro* re-stimulation with pb9 peptide **(A)**, or with MVA vector-specific peptides **(B and C)** (all three peptides are CD8^+^ T cell specific). **28 days** (middle), or **84 days** (bottom) post immunization, five mice were sacrificed and spleens collected for intracellular cytokine staining and flow cytometry to determine the percentage of IFN-γ-secreting CD8^+^ T splenocytes in response to *in vitro* re-stimulation with pb9 peptide **(A)**, or with MVA vector-specific peptides **(B and C)**. These values are presented after subtracting the values of unstimulated cells for every mouse (sample). The mean of each group with the SEM error bars are shown. Data is representative of two independent experiments. * *P* = 0.0331 using Mann Whitney test.

As there was no difference with the TIP antigen responses upon deletion of clusters of genes, we wanted to determine the long-term immune responses of a the new large deletion mutant, Δ15-MVA-TIP, measuring the response to pb9, an immunodominant peptide to allow for following the long-term memory immune responses, in addition to measuring the peak immune responses. Ten BALB/c mice, per group, were immunized with either the Δ15-MVA-TIP mutant or the control (MVA-TIP) 10^6^ pfu via i.m route, and the immune responses followed for three months. First, all mice were bled at day 7 post-immunization and the PBMCs were screened in blood-ELISpot using the pb9 peptide, or the vector-specific E3 and F2(G) peptides. The CD8^+^ T cell immune responses were similar between the MVA-TIP and the mutant (Δ15-MVA-TIP). At day 28 post-immunization, five mice from each group were sacrificed, spleen harvested, and ICS was performed using the same three peptides. Only E3-specific CD8^+^ T cell responses were enhanced (in two independent experiments) in the mutant group while there were no differences in the recombinant epitope (pb9) or in the other vector-specific F2(G) peptide. The remaining of the mice were sacrificed at day 84 post-immunization and showed no improved CD8^+^ T cell frequencies, even the increased responses with the E3 peptide at day 28 was not observed at day 84, suggesting it was transient enhancement (Fig 2).

### The immunogenicity of a recombinant MVA mutant lacking fifteen genes, with TIP antigen in DNA-prime MVA-boost vaccination

**Fig 3:**
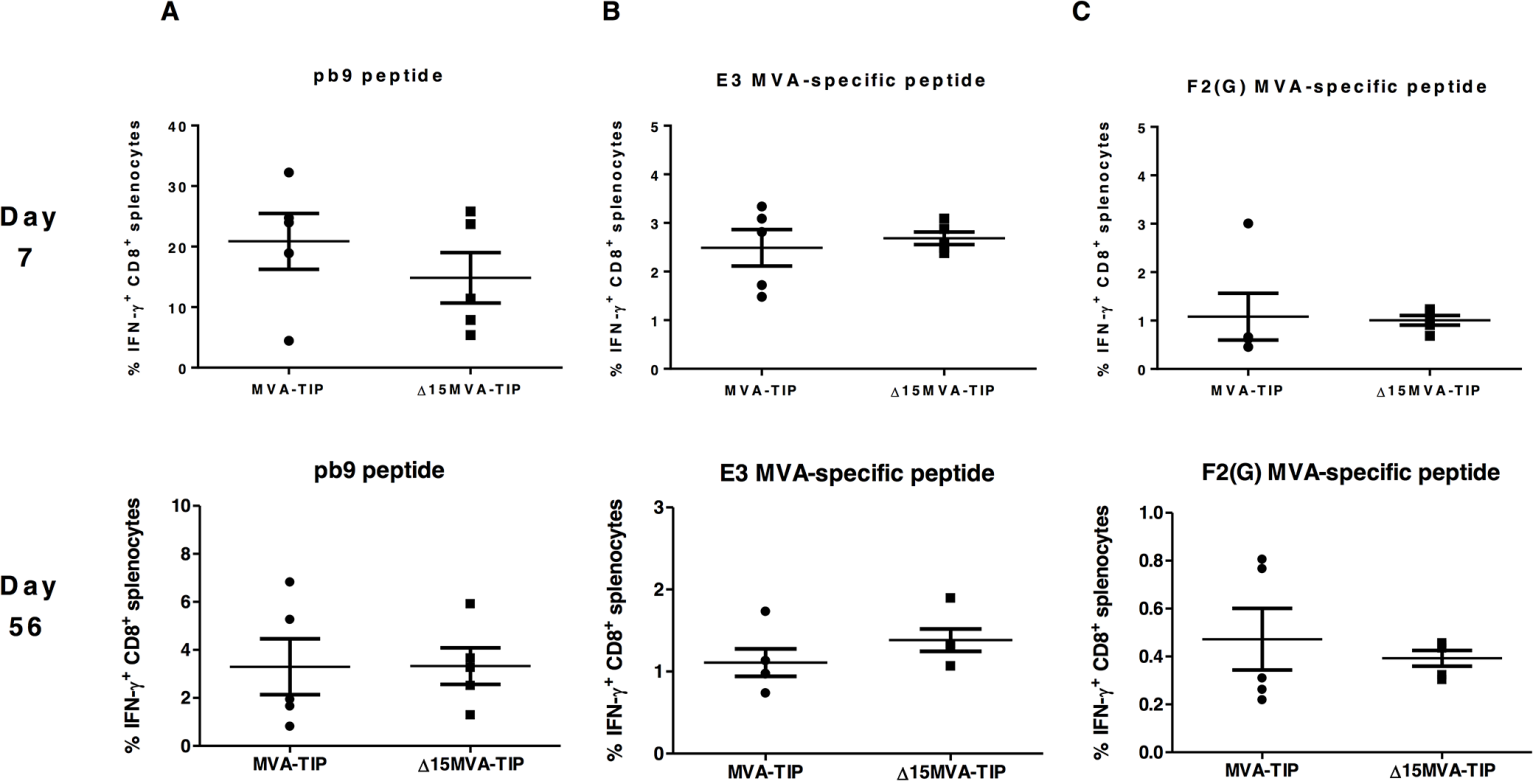
*In vivo* cellular immunogenicity of MVA deletion mutants, lacking fifteen genes, with TIP antigen, in a prime-boost regimen. Two groups of female BALB/c mice (n = 10) were immunized i.m. with 100μg of pSG-TIP DNA, then boosted with i.p. injection of MVA*wt* with TIP model antigen (MVA-TIP), or MVA deletion mutant (lacking 15 genes) ∆15-MVA-TIP at the dose of 1x10^7^ pfu/ml. **7 days** (top), or **56 days** (bottom) post-boost immunization, half of the mice were sacrificed and spleens collected for intracellular cytokine staining and flow cytometry to determine the percentage of IFN-γ-secreting CD8^+^ T splenocytes in response to *in vitro* re-stimulation with pb9 peptide **(A)**, or with MVA vector-specific peptides **(B and C)**. All values are presented after subtracting the values of unstimulated cells for every mouse (sample). The mean of each group with the SEM error bars are shown. Data is representative of two independent experiments. There was no statistical significant difference between the tested groups using Mann Whitney test.

As the fifteen-gene deletion mutant did not show any difference at peak or memory responses, we proceeded to test this mutant in the more clinically relevant prime-boost vaccination regimen. This regimen has been used to test other MVA deletion mutants (e.g. ΔA41\B15R-MVA-B [13] and ΔC6-MVA-B [14] with reported increases in immunogenicity from the mutant compared to wild type MVA. Thus, mice were primed with 100μg DNA, encoding the TIP antigen, via i.m. route, and boosted two weeks later with 10^7^ pfu, via i.p. route, with either the Δ15-MVA-TIP mutant or the control (MVA-TIP). Five mice were sacrificed at day 7 post-boost to determine the peak immune responses with the remaining mice sacrificed at day 56 post-boost to determine the memory responses. There were no improved CD8^+^ T cell responses either at the peak or at the memory responses to pb9, E3, or F2(G) peptide stimulations (Fig 3).

### The immunogenicity of a recombinant MVA mutant lacking fifteen genes, with 85A antigen

**Fig 4:**
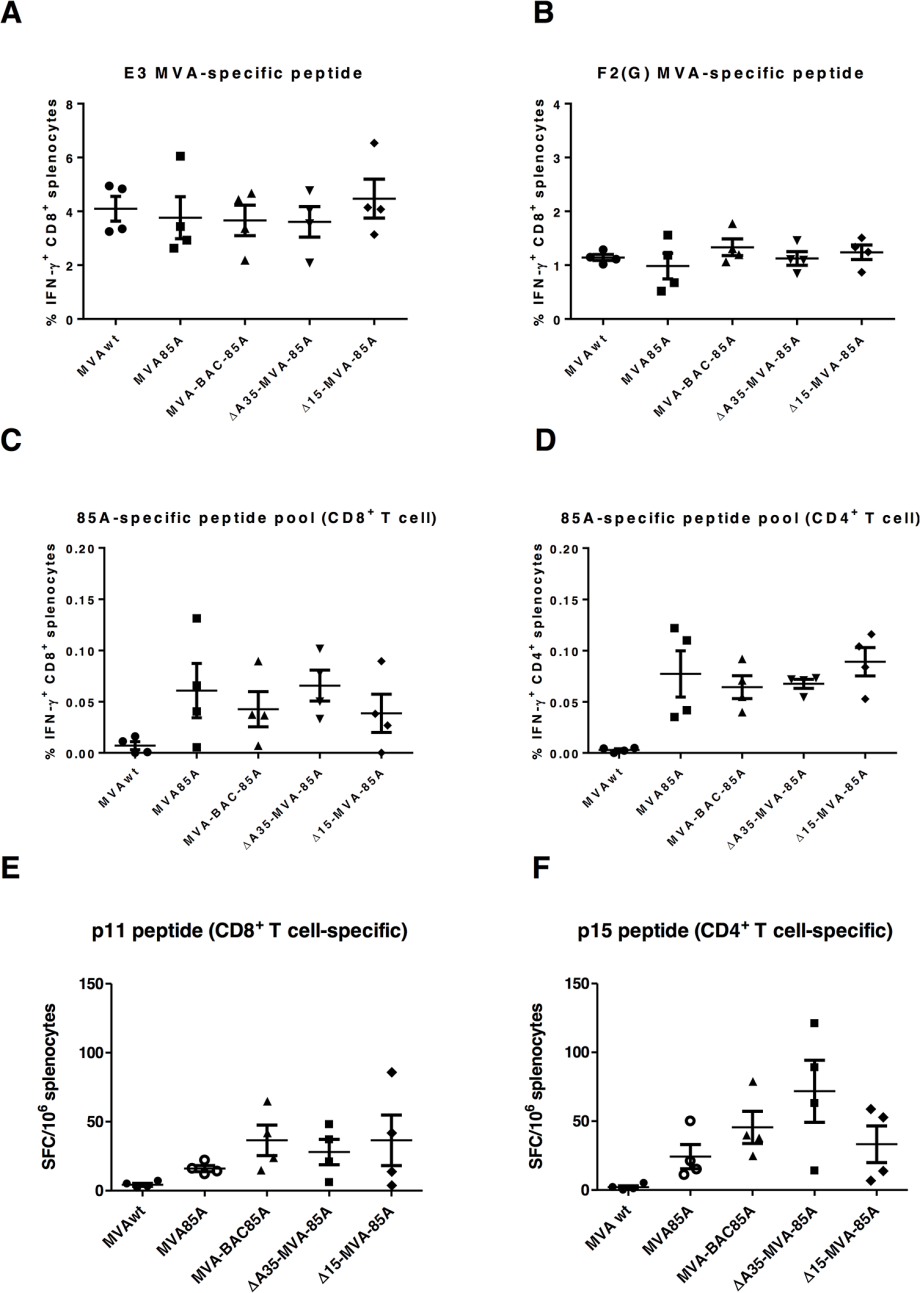
*In vivo* cellular immunogenicity of MVA deletion mutants, lacking fifteen genes, with 85A antigen. Four groups of female BALB/c mice (n = 4) were immunized (i.m.) with the respective MVAs at the dose of 2x10^6^pfu/ml. Seven days post immunization, intracellular cytokine staining and flow cytometry was performed to determine the percentage of splenic IFN-γ-secreting T cells in response to *in vitro* re-stimulation with MVA vector-specific peptide (**A** and **B**), with CD8^+^ T cell specific 85A peptide pool **(C)**, or with CD4^+^ T cell specific 85A peptide pool **(D)**. Two individual peptides were incubated for 18 hours to determine the CD8^+^ **(E)** or CD4^+^ T **(F)** cell responses by ELISpot. These values are presented after subtracting the values of unstimulated cells for every mouse (sample). The mean of each group with the SEM error bars are shown. Data is representative of two independent experiments. There was no statistical significant difference in any of MVA mutant groups as compared to MVA85A or MVA-BAC85A groups, using Kruskal-Wallis test with Dunn's multiple comparisons. **MVA*wt*:** MVA wild type, **MVA85A:** MVA expressing TB 85A antigen, **MVA-BAC85A:** MVA, containing BAC DNA and expressing TB 85A antigen, and **∆15-MVA-85A:** MVA mutant, containing BAC DNA and expressing TB 85A antigen, with 15 gene deleted (Table 1).

As there was no detectable difference in the immune responses to the TIP, which is a synthetic epitope string-based antigen, we wished to also test a naturally occurring immunogenic antigen. The *M*. *tuberculosis* Ag85A is being tested as TB vaccine candidate, delivered by MVA vector (MVA85A) and has been shown to induce CD4^+^ and CD8^+^ T cell responses and when used to boost BCG-vaccinated adult or infant humans [23]. Thus, the Δ15-MVA-85A mutant along with other three control viruses; MVA wild type (MVA*wt*), non-mutant MVA-BAC-85A, and the MVA85A (see materials and methods), were used to immunized BALB/c mice, with 10^6^ pfu via i.m route, and the spleens were harvested one week later. The intracellular cytokine staining (ICS) was performed to determine the cellular immune responses using two 85A peptide pools, specific for CD4^+^ or CD8^+^ T cells, or E3 and F2(G) MVA-peptides specific for CD8^+^ T cells. The ICS did not show any difference in the frequencies of IFN-γ secreting CD4^+^ or CD8^+^ T cells between the mutant and any of the control viruses. The comparison of MVA*wt* and the MVA-BAC-85A showed similar vector-specific immune responses using CD8^+^ T cell-specific MVA peptides (E3 and F2(G) peptides). Moreover, the conventional MVA85A induced similar anti-vector and anti-rAg85A responses as the MVA-BAC-85A. Taking together, this supports our earlier observation [22] that inserting BAC DNA into the MVA genome did not alter the immune responses to the immunodominant vector epitopes E3 and F2(G) or to the recombinant antigens. Next, we tested the immune responses using two defined strong immune epitopes (named p15 and p11) specific to CD4^+^ or CD8^+^ T cells by ELISpot and observed a similar result to the ICS (Fig 4).

## Discussion

Here we investigated the effect of deleting some immunomodulatory or unknown non-essential genes from MVA, in different combinations (Table 1), on the cellular immunogenicity. Deleting either few or many genes from MVA showed no difference in CD8^+^ T cells to the murine malaria epitope pb9 or to the vector-specific E3 and F2(G) epitopes. The use of TIP model antigen, which harbours the immunodominant pb9 epitope, enabled us to follow the immune responses for three months, but did not show any improvement at the long-term memory CD8^+^ T cell responses (for three months). Many previous studies showed that the single deletion of *C6L* [14], *N2L* [17] or *C12* [18]; or the double deletion of *A41L*/*B15R and C6L*/*K7R* from MVA vectors enhanced the cellular immunogenicity in DNA-prime MVA-boost vaccination regimens. This regimen is also relevant to MVA clinical testing applications; MVA is used as a boosting agent in many vaccine clinical trials [26]. Thus, we applied this regimen priming with DNA, via i.m. route, and boosting with MVA, given i.p. Some of these genes, like *A41L* and *B15R* are deleted in the Δ15-MVA-TIP mutant, and therefore this mutant would be expected to elicit stronger cellular immune responses, especially in this regimen as reported before. However, our result did not show any improved immunogenicity with the Δ15-MVA-TIP in prime-only or prime-boost vaccinations. Garber *et al* (2012) reported that the deletion of four immunomodulatory genes enhanced MVA cellular immunogenicity in macaques, but the deletion of a fifth gene did not extend this improved immunogenicity [20]. Yet, when this fifth gene was deleted singly, the immunogenicity was enhanced in the same experimental setting [21]. It could be that the deletion of some genes could compensate for the absence of other deleted genes and the overall immunogenicity is not improved, but the unaltered immunogenicity with the large deletion mutant (Δ15-MVA-TIP), which lacks *A41L* or *B15R*, was also noted when the *A41L* or *B15R* were singly deleted, as we previously published [22]. Moreover, these two genes were deleted in different cocktails of deleted gene-clusters in different mutants in the current study (Table 1) and none of these mutants showed any enhanced immune responses. This could rule out the possibility that the enhancement by some deletions could be rendered useless by other deleted genes of the same mutant. The deletion of *A41L* or *B15R*, or both of them was reported to enhance MVA cellular immune responses, but this enhancement was 2-fold at best [11–13]. Although there were some detailed technical or biological differences in each study concerning the deletion of *A41L* or *B15R*, or both, the inability to reproduce this improved immunogenicity could suggest that improving MVA immunogenic profiles could be very difficult to achieve through gene deletions, especially as MVA does not replicate *in vivo*, and its gene expression declines within 12 hours, and vanishes less than 48 hours post-inoculation *in vivo* [27, 28].

The concern over the use of non-natural model antigen like TIP should not apply to a “real” immunogenic antigen. The TB 85A is one of the most advanced candidates in clinical trials and showed strong CD4^+^ T and CD8^+^ T cell immunogenicity. Thus, we replaced TIP with 85A antigen in the large MVA deletion mutant (deriving Δ15-MVA-85A), confirmed that no other changes had occurred in the genome by sequencing, then tested it in mice, measuring both CD4^+^ T and CD8^+^ T cell responses with peptide pools by ICS or two strong individual peptides (specific for CD4^+^ or CD8^+^ T cells) by ELISpot. Here, three control viruses were included; the MVA85A vaccine that is used for clinical testing against TB, the MVA-BAC-85A (which is similar to MVA85A, except it was derived via MVA-BAC recombineering), and the MVA wild type (MVA*wt*), which is non-recombinant and non-mutant control. The results showed a continued lack of improved immunogenicity even with this natural immunogenic antigen. Although we were able to test CD4^+^ T cell responses, there was no significant difference in the magnitudes of 85A-specific CD4^+^ T cell between the mutant group and any of the control groups.

It could be speculated that the MVA-BAC system or deriving MVA in BHk-21 cell lines had some effect on these mutants, which could prevent them from enhancing the immune responses. The BHK-21 cell line was reported to allow for the replication of some MVA mutant lacking *E3L*, which is difficult to grow in CEFs[29]. This was because BHK-21 cell line expresses low concentration of IFN-α/β upon the infection of the E3-deleted MVA mutant, unlike CEFs that express high concentration of IFN-α/β, leading to abortive replication of the mutant [29]. The BHK-21 cells have also a low level of PKR (double-stranded RNA-dependent protein kinase), compared to the CEFs, which allowed for the growth of the E3-deleted MVA mutant [29]. The PKR is an important immune mediator in the NFκB activation, so the growth of MVA mutants in BHK-21 cells may have less immune pressure. However, the proposed effect of MVA-BAC system or BHK-21 cells could be ruled out by comparing MVA85A and MVA-BAC-85A; the MVA85A was derived in the conventional method of constructing vaccinia virus recombinants and was propagated in CEFs whereas the MVA-BAC-85A was derived using MVA-BAC bacterial system and has undergone multiple rounds of MVA-BAC recombineering, then rescued using fowlpox virus and propagated in BHK-21 cell lines. Both viruses induced similar CD4^+^ and CD8^+^ T cell responses to the rAg-specific peptide pools by ICS or to the individual strong peptides measured by ELISpot as well as similar CD8^+^ T cell responses to the vector-specific E3 and F2(G) peptides. Moreover, this was also supported by the result of MVA*wt* compared to the MVA-BAC-85A that showed similar responses to the vector specific E3 and F2(G) peptides. This MVA-BAC system was also shown to have no altering affect on the immunogenicity of MVA [22]. The MVA85A was included as a control to relate these modifications to the clinical testing as well, but we detected similar responses with MVA85A to the mutant and to the MVA-BAC-85A. In addition, the recombinant antigen-specific IFN-γ-secreting CD8^+^ T cells was the main readout as it would be more relevant for the improvement required for clinical testing of MVA-based vaccines.

The ΔC11-K3/B15-MVA lacks *C12L* and *C6L*, which were reported to enhance cellular immunogenicity when deleted from MVA [14, 18], did not show any difference in cellular immune responses. Again, the single deletion of *C12L* [22] or *C6L* (data not shown) failed to enhance the immune responses to TIP.

In conclusion, none of the derived rMVA mutants showed improved immunogenicity either to the MVA antigens or to the encoded TIP antigen. Focusing on the large deletion Δ15-MVA-TIP mutant, neither prime only nor prime-boost regimens showed any improved CD8^+^ T cell responses. The memory responses at 28 or 84 (long-term) days post-MVA immunization showed no improved memory CD8^+^ T cell frequencies, except to one peptide (E3) at day 28. At day 56, in prime-boost regimen, this increase was observed but it was very marginal and insignificant. Even replacing TIP with TB 85A “real” antigen (Δ15-MVA-85A) and testing CD8^+^ as well as CD4^+^ T cells magnitudes did not detect any differences compared to various control viruses. This study suggests that the previously reported immunogenicity increases in MVA deletion mutant, which were small increases in many cases (2-fold), may not be replicated with different antigens. The approach of gene deletion to improve MVA-vectored vaccines should be carefully assessed for every recombinant antigen, and epitope (peptide), and may not be taken as a generic approach for improving MVA immunogenicity.

## Materials and Methods

### Ethics Statement

All animal procedures were performed in accordance with the terms of the UK Animals (Scientific Procedures) Act (ASPA) for the project licenses 30/2414 or 30/2889 and were approved by the University of Oxford Animal Care and Ethical Review Committee. All mice were housed at least 7 days for settlement prior to any procedure in the University animal facility, Oxford, UK under Specific Pathogen Free (SPF) conditions.

### Recombinant antigens

All rMVA mutants express a model antigen, called TIP [24] which is string of epitopes that encodes an H2-K^d^ restricted murine CD8^+^ T cell epitope SYIPSAEKI (pb9) from the *Plasmodium berghei* circumsporozoite protein [25]. TIP was inserted into MVA-BAC first, and then the MVA-BAC containing TIP was used as a template for making the described deletions (see Table 1). In the prime-boost regimen, the TIP was also cloned into pSG2 plasmid (pSG-TIP) and given i.m. at 100μg per mouse. In one rMVA mutant, Δ15-MVA-TIP, the TIP was replaced with the *Mycobacterium tuberculosis* 85A antigen (TB 85A), deriving the Δ15-MVA-85A. The 85A was also inserted into two other viruses to serve as controls; first, into a non-mutated MVA-BAC using MVA-BAC recombineering (virus named MVA-BAC-85A); second, into MVA wild type using the conventional recombinant vaccinia virus construction (named MVA85A, a current TB vaccine candidate in clinical trials [23]).

### Construction of rMVA deletion mutants using MVA-BAC

Construction and generation of MVA-BAC and generation of rMVA deletion mutants using *GalK* recombineering [30] has been described previously [22]. To generate recombinant MVA (rMVA) viruses expressing TIP, a cassette was constructed using conventional PCR and restriction enzyme based cloning, comprising the TIP antigen, preceded by the early/late p7.5 viral promoter and the bacterial *GalK* resistance gene under a prokaryotic promoter. This was amplified with Phusion (Finnzymes) as a targeting DNA for recombineering by using long oligonucleotide primers (Eurofins MWG Operon) to add 50bp homology arms (matching two regions within the *TK* ORF) to the 5’ and 3’ ends. Primers were designed to insert the TIP-*GalK* cassette into the *TK* locus. These targeting constructs were used for MVA-BAC recombineering as previously described [22]. Next, MVA-BAC recombineering was performed to remove the *GalK* from MVA-BAC that contains TIP-*GalK*, leaving only the TIP into the *TK* locus. Therefore, *GalK* was then amplified with long oligonucleotide primers with 50 bp homology arms, matching the flanks of the targeted ORFs, deleting and replacing them with *GalK*. The colonies on *GalK*-positive plates were re-streaked three times before the recombineered MVA-BAC constructs are then confirmed by identity and purity PCR. The presence of the TIP was also confirmed by PCR and sequencing.

### MVA-BAC rescue, and propagation and titration of rMVA mutants

The recombineered MVA-BACs were rescued to recombinant MVA in BHK cells using a fowlpox virus helper as previously described [22]. The *GalK* bacterial marker gene was only removed from mutants that required a multiple deletions such as the Δ15-MVA-TIP, for the mutants with only one gene or one region of deleted genes, the *GalK* was not removed. BACs and derived viruses were checked for identity and purity by PCR and the sequences of the homology arms and transgenes were confirmed at both stages. BAC-derived rMVAs were plaque-picked three times to ensure purity, as a precautionary measure, since the bacterial colonies were re-streaked three times prior to rescue. The viruses were amplified in 1500cm^2^ of BHK cell monolayers, purified over sucrose cushions and titred in BHK-21 cells according to standard practice, and purity and identity were again verified by PCR. Since MVA-BAC has a GFP marker gene under control of the Fowlpox virus p4B promoter [22], all the rMVAs expressed GFP, which was used for plaque picking and titration, in addition to the recombinant antigens.

### Genome sequencing of the large rMVA deletion mutant, lacking fifteen genes

DNA samples of the Δ15-MVA-85A from bacterial LB culture (for MVA-BAC DNA) or from BHK-21-infected cells (for viral DNA) were isolated using EndoFree Plasmid Maxi Kit (Qiagen) or standard phenol-chloroform DNA extraction, respectively. Samples were sent to the High-Throughput Genomics Group at the Wellcome Trust Centre for Human Genetics, University of Oxford, for MVA genome sequencing.

### Peptides, spleen samples, and mouse immunogenicity

Female BALB/c mice aged 6 to 8 weeks (Harlan Laboratories, UK) were immunized intramuscularly (i.m.) in the tibialis muscles (50μL per mouse) or intradermally (i.d.) with a total of 10^6^ pfu of rMVA. For the heterologous prime boost regimen, mice were immunized with 100 μg of DNA, given i.m. (50μL per mouse) followed eight weeks later with 10^7^ pfu, given intraperitoneal (i.p.) of rMVA. Procedures were performed in accordance with the UK Animals (Scientific Procedures) Act 1986 under granted project licenses 30/2414 or 30/2889. Splenocytes were harvested seven, or 56 days post-MVA immunization for analysis by IFN-γ ELISpot or intracellular cytokine staining (ICS) and flow cytometry as previously described [22, 31], using re-stimulation with 1μg/mL Pb9 peptide [25], 1μg/mL E3 and F2(G) peptides. or the long-term immunity assessment, samples were harvested at day 7, 28, and 84 post-MVA immunization. For the 85A responses, the re-stimulation was performed using CD4^+^ or CD8^+^ T cell-specific 85A peptide pools for ICS. Two 85A-specific individual peptides were used for ELISpot; CD8^+^ T cell-specific p11 peptide, EWYDQSGLSVVMPVGGQSSF, and CD4^+^ T cell-specific p15 peptide, TFLTSELPGWLQANRHVKPT.

### Statistical analysis

GraphPad Prism (GraphPad software) was used for statistical analysis and to plot data.
